# Model systems and unique biological features of high and low-grade colorectal cancer (CRC) revealed by xenografting 84 human CRC cell lines

**DOI:** 10.1038/s42003-025-08251-0

**Published:** 2025-06-05

**Authors:** Ian Y. Luk, Jennifer K. Mooi, Dmitri Mouradov, Tao Tan, Cameron M. Scott, Fiona Chionh, Laura J. Jenkins, Camilla M. Reehorst, Rebecca Nightingale, Peter Savas, Janson WT Tse, Rebekah LI Crake, Eduard Batlle, Diego Arango, Higino Dopeso, Peter Gibbs, Niall C. Tebbutt, Rod B. Luwor, Andrew M. Scott, Faiza Basheer, Amardeep S. Dhillon, Nicholas J. Clemons, David S. Williams, Ron Firestein, Oliver M. Sieber, John M. Mariadason

**Affiliations:** 1 Olivia Newton-John Cancer Research Institute, Melbourne, Victoria Australia; 2 La Trobe University School of Cancer Medicine, Melbourne, Victoria Australia; 3 Department of Medicine, University of Melbourne, Melbourne, Victoria Australia; 4 Walter and Eliza Hall Institute, Melbourne, Victoria Australia; 5 Department of Medical Biology, The University of Melbourne, Parkville, VIC 3052 Australia; 6 Peter MacCallum Cancer Centre and Sir Peter MacCallum Department of Oncology, The University of Melbourne, Melbourne, Victoria Australia; 7 Institute for Research in Biomedicine (IRB Barcelona), The Barcelona Institute of Science and Technology (BIST), Barcelona, Spain; 8 Centro de Investigación Biomédica en Red de Cáncer (CIBERONC), Barcelona, Spain; 9 Institució Catalana de Recerca i Estudis Avançats (ICREA), Barcelona, Spain; 10 Biomedical Research Institute of Lleida, IRBLleida, Lleida, Spain; 11 Memorial Sloan-Kettering, New York, NY USA; 12 Department of Surgery, University of Melbourne, Parkville, Victoria Australia; 13 Institute of Mental and Physical Health and Clinical Translation, School of Medicine, Deakin University, Waurn Ponds, Victoria Australia; 14 Department of Pathology, Austin Health, Melbourne, Victoria Australia; 15 Centre for Cancer Research, Hudson Institute for Medical Research, Monash University, Melbourne, Victoria Australia; 16 Department of Biochemistry and Molecular Biology, Monash University, Clayton, VIC 3800 Australia

**Keywords:** Cancer, Cell biology

## Abstract

Colorectal cancers (CRCs) present across a range of differentiation grades, which impact patient outcome and management; however, the molecular features and drivers of differentiation status are not fully understood. To address this, 84 commonly used human CRC cell lines were grown as xenografts in mice, revealing models of low-grade (LG) and high-grade (HG) CRC. Transcriptional profiling revealed coordinate downregulation of multiple transcription factors involved in intestinal development and differentiation, markers of colonic lineage-specific differentiation, and effectors of normal functions of the colonic epithelium in HG tumours. Mechanistically, multiple genes suppressed in HG tumours harboured promoter methylation, indicative of stable epigenetic silencing. Furthermore, markers of LGR5+ colon stem cells were suppressed in HG tumours, while markers of cell proliferation, fetal-like intestinal stem cells, and non-canonical cell types including mesenchymal cells were increased. These changes manifested in HG cell line displaying increased proliferation, migration and metastatic capacity. Importantly, CRC cell line-derived transcriptional profiles of differentiation grade were reflected in LG and HG patient-derived tumour organoids and primary CRCs, revealing cell lines accurately model differentiation grade. The models and tumour differentiation-related properties identified herein may inform new approaches for tailored CRC treatments based on tumour grade.

## Abstract (web summary)

Colorectal cancer cell lines accurately recapitulate the histological and transcriptional properties of low-grade and high-grade colorectal cancers.

## Introduction

Poorly differentiated colorectal cancers (CRCs) have increased metastatic propensity and poorer outcome^1,2^. Studies in mouse models involving the deletion of colonic lineage-specific transcription factors have also shown that differentiation loss directly contributes to tumour development^3–5^. The maintenance of cell differentiation is therefore emerging as an important feature for preventing both the risk of tumour development as well as metastatic spread. Understanding the functional consequences of differentiation loss and the molecular events that underpin this process are key to it’s prevention and reversal, which requires access to relevant model systems.

Differentiation status, or histological grade, of conventional colorectal adenocarcinomas, is defined based on the resemblance of the tumour to the glandular architecture of the normal colonic epithelium. Low-grade (well- to moderately differentiated) CRCs (~80% of cases) largely retain glandular structures, whereas high-grade (poorly differentiated) tumours (~20%) show more solid growth patterns, with partial or complete loss of glandular structure^6,7^. In addition to conventional adenocarcinomas, several less common histological subtypes of CRC are also recognised^8^. These include mucinous, serrated, micropapillary and adenoma-like adenocarcinoma, signet-ring cell, medullary and adenosquamous carcinoma, as well as carcinomas with sarcomatoid components, undifferentiated carcinoma and neuroendocrine carcinoma, which by definition are poorly differentiated^7,9^. Poorly differentiated adenocarcinomas, as well as a number of these poorly differentiated histological subtypes (mucinous adenocarcinoma, signet-ring cell carcinoma, medullary carcinoma), are associated with microsatellite instability (MSI)^10^, *BRAF* mutations and the CpG island methylator phenotype (CIMP)^11^. While the association between tumour grade and outcome in CRC is somewhat confounded by these molecular associations^9^, higher tumour grade is nevertheless associated with poorer outcome in stage III CRC cohorts adjusted for MSI status^12^, and high tumour grade is one of the high-risk features that may be used to recommend patients for adjuvant chemotherapy^13^.

In addition to the differences in histology, at the cellular level, loss of differentiation involves loss of cell-cell contacts^14^ and cell polarity^15^, while at the molecular level, poorly differentiated tumours display reduced expression of drivers (e.g., CDX2)^16^ and markers (e.g., VIL1 and KRT20)^17,18^ of colonic differentiation. However, other transcriptional differences between high and low-grade tumours and the underlying mechanisms which drive these differences remain poorly characterised. In this regard, studying the features and molecular drivers of differentiation loss using heterogeneous primary tumour specimens is confounded by the presence of multiple cell types, including stromal, immune and endothelial cells within these specimens. CRC cell lines therefore represent a powerful tool for studying the molecular basis of differentiation loss in CRC, and for the identification and testing of new treatments^19^, including those that could preferentially target the more aggressive HG subset.

Comprehensive genomic^19,20^, transcriptomic^21^ and proteomic^22^ profiling of CRC cell lines by us and others has shown that despite extensive culture in vitro, cell lines retain the major molecular alterations observed in primary tumours^19,20^, establishing them as robust disease models. However, whether cell lines accurately model the different histological subtypes of CRC has not been fully characterised.

To address this and establish a resource for researchers in the field, we undertook a comprehensive histopathological analysis of xenografts generated from 84 commonly used CRC cell lines, which we had previously characterised at the genomic level^20^. Histological analysis of the resulting xenografts revealed models of low and high-grade tumours. Gene expression profiling revealed that key colon developmental transcription factors and markers are downregulated in HG CRC cell lines, as are genes serving major functions of the normal colonic epithelium, such as fatty acid metabolism and xenobiotic detoxification. Strikingly, a high proportion of genes suppressed in HG tumours displayed increased promoter methylation, consistent with stable epigenetic silencing. Notably, HG CRC cell lines also lose expression of markers of LGR5+ colonic stem cells, but instead gain markers of cell proliferation, fetal-like intestinal stem cells, and non-canonical cell types, including mesenchymal cells, which was reflected in increased proliferative and migratory capacities of these cell lines.

Importantly, genes differentially expressed between LG and HG CRC cell lines grown in culture were retained when cell lines were grown as xenografts, and were broadly reflected in LG vs HG patient-derived tumour organoids and primary CRCs. Collectively, these findings demonstrate that CRC cell lines accurately model the spectrum of differentiation states observed in CRC, and reveal a robust transcriptional signature associated with differentiation loss.

## Results

### Growth of 84 colorectal cancer cell lines as xenografts

**Fig. 1:**
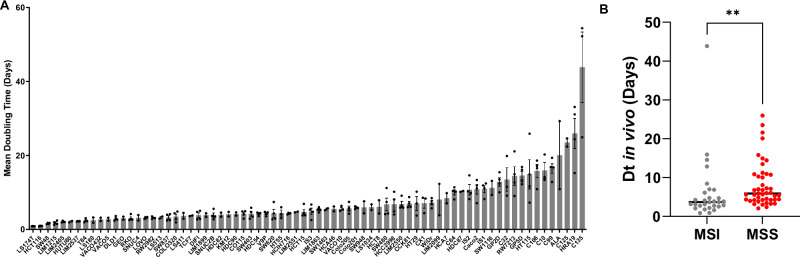
Growth of 84 CRC cell lines as xenografts. **A** Doubling time of 74 (of 84) CRC cell lines grown as xenografts in Balb/*c nu/nu* mice. Balb/*c nu/nu* mice were injected with 2 × 10^6^ cells for each cell line in the right and left flanks, and tumour growth was monitored every 2 or 3 days by caliper measurement. Data shown are the mean doubling time (Dt) and standard error (SEM) of *n* = 2–4 tumours per cell line for which Dt could be computed (*n* = 74). **B** Doubling times of cell lines separated according to MSS (*n* = 45) or MSI status (*n* = 29). Lines represent the mean of each group, and groups were compared using a non-parametric Mann–Whitney *t*-test, **P < 0.005.

To identify models of high and low-grade CRC, 84 commonly used CRC cell lines were grown as xenografts in immunocompromised Balb/*c nu/nu* mice, of which 82 (98%) successfully formed tumours. The 2 cell lines that failed to grow in Balb/*c nu/nu* mice could be successfully grown in NOD*-scid* IL2Rgamma-c^null^ (NSG) mice, a model of further immune compromise. Tumour doubling time (Dt) could be computed for 74 or the 82 cell lines that grew in Balb/*c nu/nu* mice, which ranged widely from <1 day for the fastest growing lines (LS174T, HCT116 and SW48) to >25 days for the slowest growing lines (C125, HRA19 and C135) (Fig. 1A) (Table S1). Notably, MSI tumours had a significantly faster growth rate compared to MSS tumours (median Dt 3.7 vs 5.8 days, *P* = 0.0016) (Fig. 1B).

### Differentiation grade of CRC cell line xenografts

**Fig. 2:**
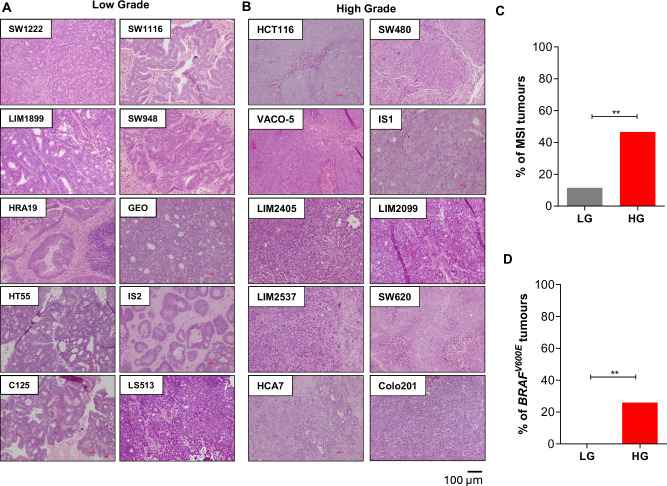
Differentiation grade of CRC cell line xenografts. **A**, **B** Representative H&E images of **A** low-grade (LG) and **B** high-grade (HG) CRC cell line xenografts. **C**, **D** Association of differentiation grade with **C** MSI status and **B** *BRAF* mutation status. **P < 0.005, Fisher’s exact test.

Once tumours reached 1 cm^3^ in size or had been growing for 3 months, they were resected and histopathologically graded by an anatomical pathologist based on the abundance of gland formation, utilising standard WHO criteria. Of the 84 xenograft tumours, 3 (3.6%) were graded as well-differentiated; 23 (27.4%) were graded as moderately differentiated; 19 (22.6%) were graded as poorly differentiated; and 39 (46.4%) were graded as undifferentiated (Table S1). Well and moderately differentiated tumours were subsequently grouped together as low-grade (LG), and poorly differentiated and undifferentiated tumours were grouped together as high-grade (HG). Tumours were then independently graded by a second anatomical pathologist, with 92% concordance observed. Tumours with discordant grades between two pathologists were subsequently resolved by a third assessor. Overall, 58/84 (69.0%) of xenografts were classified as HG and 26/84 (31.0%) were classified as LG (Fig. 2A, B; Table S1). Consistent with observations in primary disease, high tumour grade in cell lines was associated with microsatellite instability, with 46.6% of HG cell lines harbouring MSI compared to 11.5% of LG cases (*P* = 0.003 Fisher’s exact test); and with *BRAF*^*V600E*^ mutation status, with 25.9% of HG cell lines harbouring *BRAF*^*V600E*^ mutations compared to 0.0% of LG cell lines (*P* = 0.004, Fisher’s exact test) (Fig. 2C, D; Table S1). No xenografts containing signet-ring, medullary or serrated features were observed, and no xenografts had the abundance of mucin required to meet the WHO definition of mucinous CRC (>50% of the tumour).

### HG CRC cell lines lose expression of transcriptional drivers of colonic development and differentiation, and colonic lineage markers

**Fig. 3:**
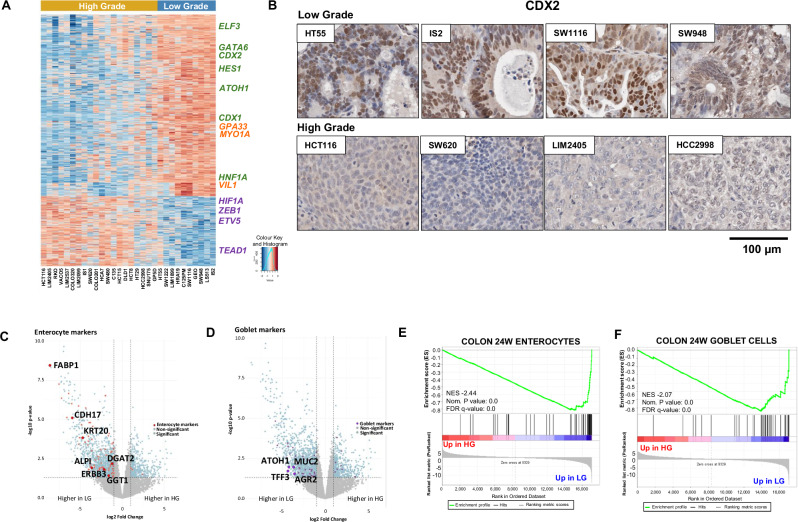
Differential gene expression between low-grade and high-grade CRC cell lines. **A** Heatmap of 1763 differentially expressed genes between high-grade (*n* = 20) (orange bar) and low-grade (*n* = 10) CRC cell lines (blue bar), highlighting differential expression of general markers of colonic epithelial cells (orange text); transcription factors involved in intestinal development and differentiation (green); and transcription factors involved in epithelial-to-mesenchymal transition (purple). **B** Immunohistochemical staining for CDX2 in *n* = 4 representative low-grade and high-grade CRC cell line xenografts. **C**, **D** Volcano plots showing higher expression of **C** enterocyte and **D** goblet cell markers in LG CRC cell lines. **E**, **F** Cell type signature GSEA showing significant enrichment of enterocyte and goblet cell markers previously identified by Gao et al.^24^ in LG tumours.

Having classified LG and HG CRC cell lines, we next sought to identify genes differentially expressed between these histological subtypes by interrogating gene expression and proteomic data previously generated for 30 of these cell lines grown in vitro^20,22^. This analysis revealed 1763 differentially expressed genes (DEGs) between LG (*n* = 10) and HG CRC (*n* = 20) cell lines (Fig. 3A), of which 1270 were more highly expressed in LG lines and 493 were more highly expressed in HG lines. This differentiation-associated gene signature was independently validated in RNAseq data from 13 overlapping CRC cell lines (*N* = 4 LG, *n* = 9 HG) profiled by the Cancer cell line encyclopedia (CCLE) project^23^ (Fig. S1A), with a strong statistically significant correlation in differential gene expression between LG and HG cell lines observed between the 2 datasets (Fig. S1C).

To determine if the differentiation-associated gene signature was retained at the protein level, we interrogated corresponding protein expression of the differentially expressed genes by analysing published proteomic profiles of these cell lines^22^. Protein expression data were available for 89 of the 1763 DEGs, which was sufficient to accurately cluster the cell lines according to grade (Fig. S1B). Furthermore, a significant correlation was observed in differential mRNA and protein expression between the LG and HG cell lines (Fig. S1D).

Detailed inspection of the differentially expressed genes further revealed that HG CRC cell lines display significantly lower expression of multiple transcription factors implicated in normal colonic development and differentiation, including *CDX1*, *CDX2*, *GATA6*, *ELF3*, *HNF4A*, *HNF1A*, *ATOH1 and HES1* (Fig. 3A), several of which were confirmed by qPCR (Fig. S2A). To further confirm this finding, 79 of the 84 xenografts for which sufficient tissue was available were stained for CDX2 expression. Of the LG tumours 23/24 (95.8%) stained positively for CDX2 (e.g., HT55, IS2, SW1116 and SW948). Comparatively, a lower proportion (35/55, 63.6%) of HG tumours stained positively for CDX2, while 36.4% of HG tumours (e.g., HCT116, SW620, LIM2405 and HCC2998) were negative for CDX2 expression (*P* < 0.0001, Fisher’s exact test) (Fig. 3B).

**Fig. 4:**
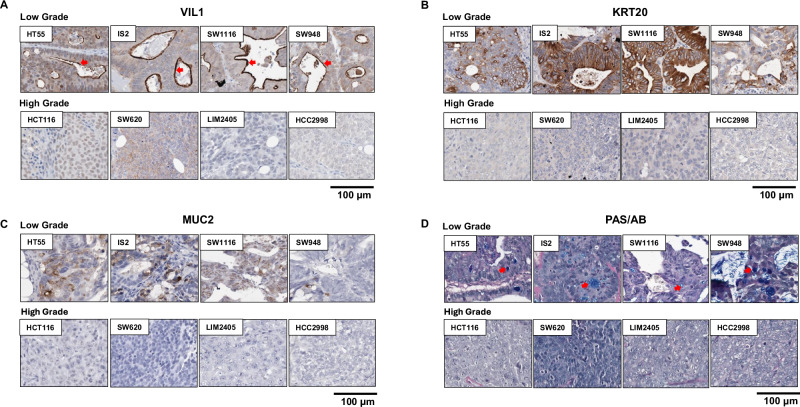
HG CRC cell lines lose expression of markers of colonic epithelial cells, enterocyte and goblet cells. **A**, **B** Immunohistochemical staining of the **A** colonic epithelial marker Villin (VIL1); **B** the enterocyte marker KRT20; and **C**, **D** the goblet cell markers **C** MUC2 and **D** PAS/Alcian Blue (AB) in *n* = 4 representative low-grade and *n* = 4 representative high-grade CRC cell line xenografts.

In line with the loss of transcriptional drivers of colonic differentiation, expression of general markers of colonic epithelial cells (*VIL1*, *GPA33*, *MYO1A*) (Fig. 3A), and markers of the major colonic cell lineages, enterocytes (*FABP1*, *CDH17*, *KRT20*, *ALPI*, *ERBB3*) and goblet cells (*TFF3*, *MUC2*, *AGR2*), were significantly downregulated in HG tumours (Fig. 3C, D). This was also confirmed by gene set enrichment analysis (GSEA) by matching to cell type-specific transcriptional signatures derived from the normal human colon (Fig. 3E, F)^24^. To confirm these changes by IHC, xenografts were stained for VIL1 and KRT20. Of the LG xenografts, 79% and 78% displayed positive staining for VIL1 and KRT20, respectively, compared to 12% and 33% of HG tumours (*P* < 0.001 and *P* < 0.0001, for VIL1 and KRT20 respectively, Fisher’s exact test) (Table S1, Fig. 4A, B). To assess markers of goblet cell differentiation, xenografts were stained with periodic acid Schiff/Alcian blue (PAS/AB) as well as for MUC2 expression by IHC. Of the LG xenografts, 73% stained positive for both goblet cell markers (positive PAS/AB and MUC2) compared to only 20% of HG xenografts (Table S1, Fig. 4C, D, *P* < 0.0001, Fisher’s exact test). Notably, of the cell lines that expressed both enterocyte (KRT20) and goblet cell markers, 10/11 (90.9%) were LG, consistent with the potential of LG tumours to give rise to both colonic lineages, whereas of the cell lines negative for all 4 markers, 14/14 were HG.

Finally, to identify cell lines with neuroendocrine features, cell blocks of the cell lines were stained for the neuroendocrine markers, synaptophysin and chromogranin. We identified one cell line, NCI-H716, with strong expression of both markers (Fig. S2B, C). This cell line originated from the ascitic fluid of a 33-year-old patient with a poorly differentiated caecal tumour that expressed neuroendocrine markers^25,26^. Another cell line, Colo320, reported to be derived from a neuroendocrine (carcinoid) colonic tumour^27^, also showed positive staining for synaptophysin, while several other lines (e.g., T84, Caco-2 and VACO10) harboured rare cells positive for either chromogranin or synaptophysin (Fig. S2B, C, Table S1). Overall, however, most CRC cell lines (94%) lacked expression of both of these neuroendocrine markers (e.g., HCT116 in Fig. S2D, E, Table S1).

### HG CRC cell lines lose expression of genes involved in normal colonocyte functions

To identify additional distinguishing features of LG and HG CRCs, we performed *Hallmark* GSEA of genes differentially expressed between HG and LG cell lines. Expression of genes involved in normal colonocyte functioning, including bile acid reabsorption^28^ (*biosynthesis of bile acids*), xenobiotic detoxification^29,30^, (*metabolism of xenobiotics*), and fatty acid metabolism^31^ (*oxidative phosphorylation* and *fatty acid metabolism*), were all downregulated in HG cell lines, indicating that HG cell lines lose these functional characteristics of normal colonocytes (Fig. S3A).

### HG CRC cell lines lose expression of markers of LGR5+ colon stem cells and gain expression of markers of fetal-like intestinal stem cells

**Fig. 5:**
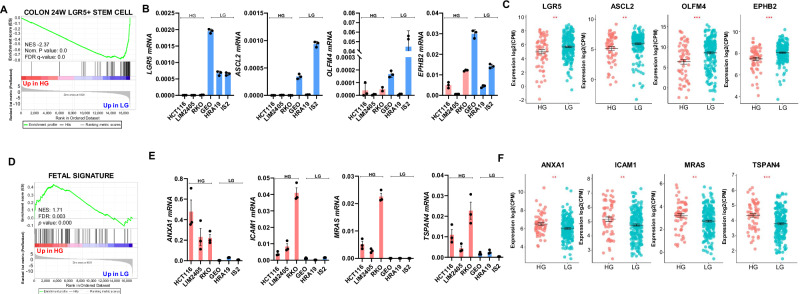
HG CRC cell lines lose expression of markers of LGR5+ colon stem cells and gain expression of markers of fetal-like intestinal stem cells. **A** Downregulation of LGR5+ colon stem cell signature in HG CRC cell lines. **B** Expression (mRNA) of LGR5+ colonic stem cell markers in a subset of HG and LG CRC cell lines determined by qPCR. Values shown are mean + SEM of a representative experiment performed in technical triplicate. **C** Expression of corresponding LGR5+ colonic stem cell markers in HG (*n* = 58) vs LG (*n* = 202) primary CRCs in the TCGA cohort (RNAseq data). **D** Enrichment of fetal-like intestinal stem cell signature^33^ in HG CRC cell lines. **E** mRNA expression of fetal-like stem cell markers in HG and LG CRC cell lines determined by qPCR. Values shown are mean + SEM of a representative experiment performed in technical triplicate. **F** Corresponding mRNA expression of fetal-like intestinal stem cell markers in HG (*n* = 58) vs LG (*n* = 202) primary CRCs in the TCGA cohort (RNAseq data). Values shown in (**C** and **F**) are mean + SEM, and groups were compared using unpaired *t*-tests. **P < 0.01, ***P < 0.005, ****P < 0.001.

Interestingly, GSEA also revealed that HG CRC lines had significantly reduced expression of markers of LGR5+ colon stem cells (Fig. 5A). This was particularly pronounced in a subset of undifferentiated MSI cell lines (RKO, HCT116 and LIM2405) which expressed very low levels of *LGR5*, *EPHB2*, *ASCL2* and *OLFM4* measured by qPCR (Fig. 5B). Importantly, reduced mRNA expression of *LGR5*, *OLFM4*, *ASCL2* and *EPHB2* was also observed in HG primary tumours in the TCGA dataset (Fig. 5C).

Replacement of normal LGR5+ colon stem cells with fetal-like intestinal stem cells occurs during regeneration of the normal colonic epithelium following injury in mice^32^, while loss of LGR5+ stem cell markers and gain of fetal-like intestinal stem cell markers have been observed during metastasis and following chemotherapy treatment in human CRC^33,34^. Indeed, examination of the fetal-like stem cell signature revealed significant enrichment in HG CRC cell lines (Fig. 5D). Validation of this finding by qPCR revealed significantly increased expression of the key markers *ANXA1*, *ICAM1*, *MRAS* and *TSPAN4* in HG cell lines (Fig. 5E), which was also confirmed in HG primary CRCs in the TCGA dataset (Fig. 5F).

It was also recently reported that increased expression of the fetal gene signature is associated with expression of non-canonical gene signatures (Injury repair, neuroendocrine and osteoblast) during colorectal cancer metastasis, with concomitant reduction in canonical gene signatures (Absorptive Intestine, Secretory intestine and Tumour ISC-like)^33^. Consistent with our earlier findings (Fig. 3), loss of the “canonical signature” was observed in HG cell lines (enriched in LG lines); however, no significant enrichment of the “non-canonical” signature was observed in the HG cell lines grown in vitro (Fig. S4A). Notably, enrichment of non-canonical gene signatures was not observed in CRC liver metastases grown as organoids, indicating their expression is primarily induced in vivo^33^. To test this, RNAseq analysis was performed on 4 LG and 4 HG cell lines grown as xenografts. This analysis revealed that while the canonical signature continued to be enriched in LG xenografts, significant enrichment of the non-canonical gene signature was now observed in HG cell line xenografts (Fig. S4B), confirming this signature predominantly manifests in vivo.

Finally, to validate this finding in primary CRCs, we investigated the association between differentiation grade and expression of the canonical and non-canonical signatures in the TCGA cohort. Consistent with the findings in cell line xenografts, significant loss of canonical signatures and gain of non-canonical signatures were observed in HG CRCs (Fig. S4C), revealing that the progressive plasticity observed during metastasis is also a feature of differentiation loss.

### HG CRCs display increased expression of genes involved in cell proliferation and epithelial-to-mesenchymal transition

GSEA analysis of the major biological hallmarks also revealed significant enrichment of genes involved in *cell cycle progression: E2F targets*, *G2/M checkpoint* and *mitotic spindle assembly* in HG cell lines (Fig. S3A). GSEA matching to normal human colon cell type signature gene sets further revealed that HG tumours resembled “*Ki67 high*” cells in the adult colon (Fig. S3B)^24^, collectively suggesting increased proliferative capacity of HG cell lines. Consistent with these findings, HG tumours had significantly shorter doubling times compared to LG tumours in vivo (median Dt 4.2 vs 6.0 days, respectively, *P* = 0.016) (Figs. 1A, S3C).

**Fig. 6:**
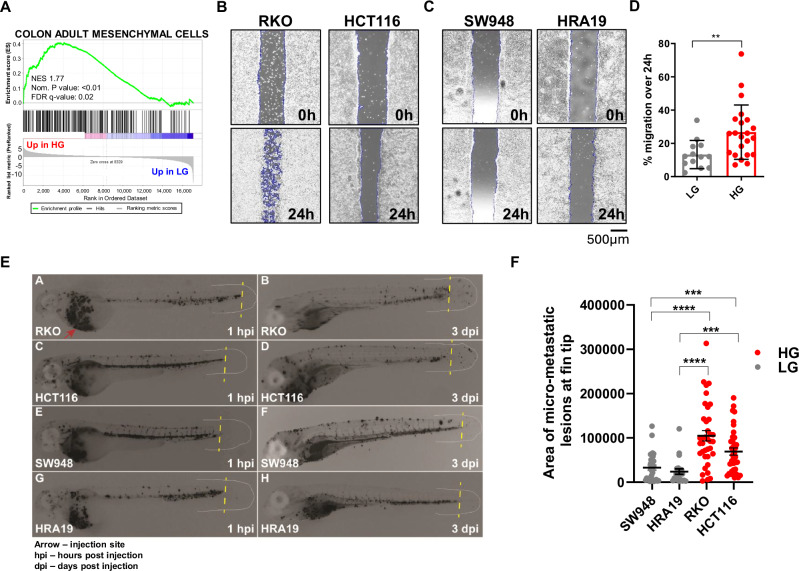
HG CRC cell lines upregulate cell proliferation genes and EMT markers. **A** Cell type signature GSEA showing significant enrichment of the “mesenchymal” gene signature previously identified by Gao et al.^24^ in HG CRC cell lines. **B**, **C** Images of cell migration over 24 h of representative **B** high and **C** low-grade cell lines. **D** Quantitation of the cell migration rates of LG (*n* = 13) and HG (*n* = 22) CRC cell lines, assessed over 24 h in vitro. Data shown are mean ± SEM. **E** Representative images of metastasis of *n* = 2 low-grade and *n* = 2 high-grade CRC cell lines in a zebrafish model in vivo. Cells were injected into the perivitelline space of zebrafish embryos and seeding of cells in the avascular fin tip (**F**) quantified after 3 days. Data shown in (**F**) are mean ± SEM of *n* = 21–37 zebrafish embryos per cell line. In all cases, groups were compared using unpaired *t*-tests. **P < 0.01, ***P < 0.005, ****P < 0.001.

GSEA also revealed that HG tumours acquire features of mesenchymal cells in the adult colon, suggestive of at least partial epithelial-to-mesenchymal transition (EMT) (Fig. 6A). In line with this phenotypic change, mRNA expression of several transcriptional drivers (*ZEB1*^35^, *HIF1A*^36^) and markers (*FSCN1*^37^, *FLNA*^38^) of EMT were increased in HG tumours (Table S1, Fig. 3A). Reduced protein expression of the epithelial marker E-Cadherin and increased protein expression of the mesenchymal marker vimentin were also confirmed in a subset of HG cell lines (Fig. S5). As EMT has been implicated in increased migratory and metastatic capacity, we first assessed the migration rates of 13 LG and 22 HG lines in vitro, which revealed significantly faster migration rates of HG cell lines (Fig. 6B–D). We further tested this phenotype in a subset of cell lines in a zebrafish model in vivo, which confirmed that the HG cell lines tested have an increased migratory and metastatic capacity as demonstrated by the higher number of increased micro-metastatic lesions formed at the avascular fin tip 3 days following injection into the perivitelline space of zebrafish embryos (Fig. 6E, F).

### Loss of differentiation markers in HG CRC cell lines is associated with increased promoter methylation

**Fig. 7:**
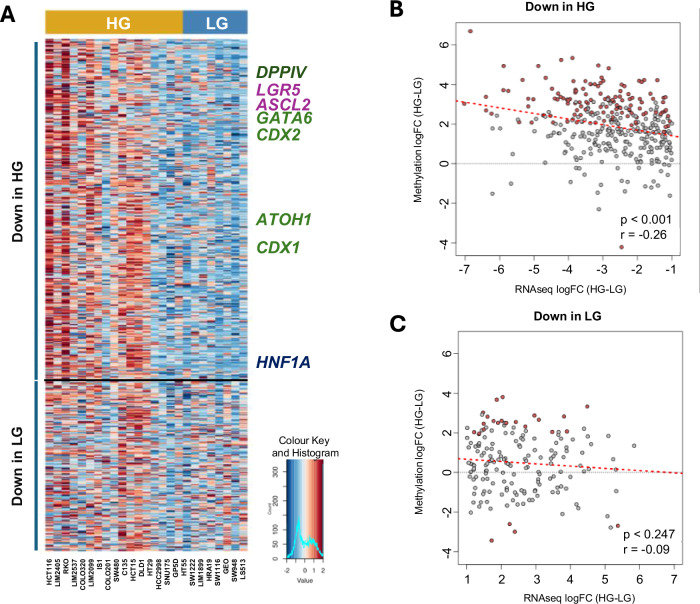
Loss of differentiation markers in HG CRC cell lines is associated with promoter methylation. **A** Heatmap of corresponding methylation changes in promoters of 519 of the 1763 genes differentially expressed between HG and LG CRC cell lines. **B**, **C** Correlation of differential promoter methylation and differential gene expression for genes with **B** lower expression in HG cell lines (*n* = 346) and **C** lower expression in LG cell lines (*n* = 173).

Next, to investigate the mechanisms driving the transcriptional reprogramming in HG CRCs, we examined corresponding methylation changes in the promoters of the genes differentially expressed between HG and LG cell lines. CpG islands were present in 519 of the 1763 DEGs, and correlation of the differences in gene expression with that of promoter methylation revealed a significant inverse correlation overall for the genes downregulated in HG tumours (Fig. 7A top, 7B). Specific genes downregulated in HG tumours which showed significantly higher promoter methylation included the developmental transcription factors *CDX1*, *CDX2*, *GATA6*, *HNF1A*, the marker of colonic differentiation, *DPP4*, and the markers of LGR5+ intestinal stem cells, *ASCL2*, *LGR5* (Fig. 7A). Notably, no overall association between gene expression and promoter methylation was observed for the subset of genes downregulated in LG tumours (Fig. 7A bottom, 7C).

### Transcriptional differences between HG and LG CRC cell lines are retained when grown as xenografts and are reflected in HG vs LG patient-derived tumour organoids (PDTOs) and primary CRCs

**Fig. 8:**
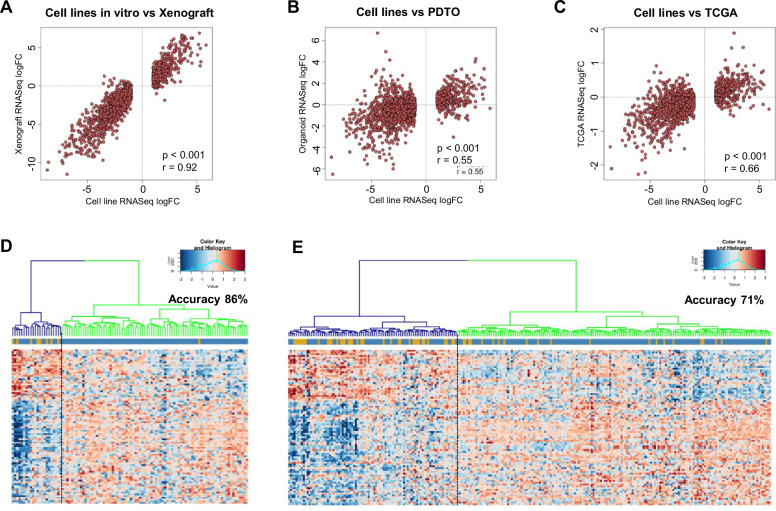
Transcriptional differences between HG and LG CRC cell lines grown in vitro are retained in xenografts, PDTOs and primary CRCs. **A**–**C** Pearson correlations of the magnitude of differential expression of genes between HG and LG cell lines (logFC) with the corresponding change in expression between HG versus LG **A** xenografts, **B** PDTOs and **C** TCGA cases. **D**, **E** Canberra clustering of **D** *n* = 95 LG and *n* = 9 HG transcriptionally profiled patient-derived tumour organoids^39^, and **E** *n* = 202 LG and *n* = 58 HG primary CRCs profiled by the TCGA, based on a 90 gene signature derived from the 1763 DEGs in cell lines using an elastic net approach.

As the transcriptional signature distinguishing HG and LG cell lines described above (Fig. 3A) was generated from CRC cell lines cultured in vitro, we next sought to determine if these transcriptional differences were retained when cell lines were grown as xenografts. Correlation of the magnitude of differential expression of the differentiation signature genes derived in vitro (*n* = 1763) with corresponding changes in a subset of the LG and HG cell lines grown as xenografts revealed a highly significant correlation (*r* = 0.92, *P* < 0.001, Fig. 8A) confirming differentiation-related transcriptional differences observed in vitro are largely retained when the cell lines are grown in vivo.

We next determined if the genes differentially expressed between LG and HG CRC cell lines displayed similar differential expression between patient-derived tumour organoids (PDTOs) derived from CRC patients with HG and LG tumours^39^. Correlation of the differential expression of the cell line-derived differentiation signature genes with corresponding changes in LG vs HG PDTOs (*n* = 104) revealed a statistically significant correlation (*r* = 0.55, *P* < 0.001, Fig. 8B), demonstrating that the cell line-derived differentiation signature is largely retained in PDTOs.

Finally, we determined whether the genes differentially expressed between LG and HG CRC cell lines were also reflected in LG and HG primary CRCs. To address this, we extrapolated the differentiation grade of 260 CRCs transcriptionally profiled by the TCGA from the associated pathology reports. Of the 1763 DEGs identified in HG vs LG CRC cell lines, 1666 were identified in the TCGA dataset. Correlation of the differential expression of the cell line-derived differentiation signature genes with corresponding changes in LG vs HG primary CRCs revealed a statistically significant correlation (*r* = 0.66, *P* < 0.001, Fig. 8C), demonstrating that the cell line-derived differentiation signature is largely retained in primary tumours.

To extend this, we next determined the capacity of the cell line-derived differentiation signature to predict the differentiation grade of PDTOs and primary tumours (TCGA cases). For this, we used an elastic net regularised regression approach (glmnet), incorporating cross-validation (cv.glmnet) and regularised regression (glmnet), to develop a refined list of 90 genes (from the original 1763 DEGs) (Supplementary Data 1). Canberra clustering was then used to dichotomise the PDTOs and TCGA cases into 2 clusters based on expression of this 90 gene signature. We found that this signature predicted differentiation grade of PDTOs with an overall accuracy of 86%, with a sensitivity of 89% and specificity of 85% (Fig. 8D). Similarly, the elastic net model correctly predicted differentiation grade of the TCGA cases with an overall accuracy of 71%, with a sensitivity of 64% and sensitivity of 73% (Fig. 8E).

## Discussion

Poorly differentiated histology (high tumour grade) is associated with poorer clinical outcome in CRC^1,2^; however, our understanding of the molecular drivers of high tumour grade is currently limited. Defining these events could inform strategies for re-inducing differentiation and attenuating differentiation-associated processes such as metastasis, and potentially reveal differentiation-related therapeutic vulnerabilities. This requires accurate models of these distinct histological subsets.

Herein, by growth and characterisation of 84 commonly used CRC cancer cell lines as xenografts, we identify multiple models of high and low-grade CRCs using standard WHO grading criteria. As observed clinically^10,11^, HG CRC cell lines identified in our study were associated with MSI and *BRAF* mutations. Of note, our analysis revealed that HG tumours are over-represented in CRC cell lines (69%), compared to primary CRCs (~20%)^6,7^, which is also in line with the over-representation of MSI lines in the cell line panel (36% compared to 10–15% of sporadic cases)^10^. The over-representation of HG/MSI tumours in established CRC cell lines may reflect their greater capacity to adapt to growth in culture; however, whether this is driven by the hypermutator phenotype, loss of differentiation, or both, remains to be determined. Interestingly, despite the improved clinical prognosis of early-stage MSI cancers^40,41^, we noted that MSI CRC cell lines grew significantly faster than MSS lines as xenografts. This likely reflects the key role of the immune system in keeping these tumours in check, and the release of their inherent proliferative capacity when grown under immunocompromised conditions. This is also supported by the pre-immunotherapy era observation that the improved prognosis of MSI tumours evident in early-stage disease is less pronounced or lost in the metastatic setting^41,42^, where these tumours have likely gained the ability to evade immune detection^43^.

Our identification of high and low-grade CRC cell lines enabled the subsequent delineation of transcriptomic differences between these histological subsets in the absence of contaminating stromal signatures. Importantly, the transcriptional differences observed between LG and HG cell lines grown in vitro were largely retained when these lines were grown as xenografts, and were able to distinguish low and high-grade primary CRCs with reasonable accuracy, indicating the molecular features associated with differentiation loss in CRC cell lines accurately model’s differentiation loss in vivo. We also observed that transcriptional differences between low and high-grade cell lines were largely retained in epithelial enriched patient-derived tumour organoids, an increasingly used model of CRC^39^.

A key finding of this study was that genes suppressed in HG CRC cell lines were associated with functions typically performed by differentiated colonic epithelial cells, including genes involved in xenobiotic detoxification and lipid metabolism. The loss of expression of xenobiotic detoxifying enzymes in HG CRCs may result in their diminished capacity to evade the cytotoxic effects of specific therapeutics and unveil previously unknown vulnerabilities. These findings highlight the potential of evaluating tumour grade in predictive biomarker studies, a feature that is not routinely reported.

In addition to the loss of markers of differentiated colonic epithelial cells, somewhat unexpectedly, HG CRC cell lines and primary tumours expressed lower levels of markers of LGR5+ colon stem cells. Notably, these findings align with prior observations, including the reduced expression of LGR5^44^, EPHB2^45^ and OLFM4^46^ in HG human CRCs; and animal studies which have shown that ablation of LGR5+ cells within colon tumours fails to induce tumour regression^47^.

Reversion of normal adult LGR5+ stem cells to a YAP1-driven intestinal fetal-like stem cell state^32^ occurs during regeneration of the normal colonic epithelium following injury in mice^48–51^. Likewise, reduced expression of LGR5+ markers and increased expression of fetal-like intestinal stem cell markers have been reported to occur during metastasis^33^, in human CRCs treated with chemotherapy^34^, and in a subset of predominantly CMS4 CRCs, where it was associated with poorer outcome^34^. Herein, we also report that loss of markers of LGR5+ stem cells is associated with a compensatory enrichment of the fetal-like intestinal stem cell signature in HG CRC cell lines and primary tumours, identifying a further context in which this reprogramming occurs. Whether this reprogramming is driven by YAP/TEAD-mediated transcription remains to be determined, although the increased expression of TEAD1 observed in HG cell lines supports this possibility. Finally, induction of fetal genes has been linked to subsequent expression of non-canonical gene programs during colorectal cancer metastasis^33^. In line with this finding, enrichment of non-canonical genes was also observed in HG CRC cell lines and primary CRCs, revealing that the progressive plasticity observed during metastasis is also a feature of differentiation loss. Notably, as reported in tumour organoids^33^, induction of non-canonical genes was predominantly observed when cell lines were grown in vivo, revealing a limitation of current in vitro culture techniques.

HG CRC cell lines were also enriched for transcriptional signatures associated with proliferating cells in the normal colon (Ki67 high), as well as transcriptional signatures associated with mesenchymal cells^24^, which manifested in reduced doubling times and increased rates of cell migration and metastasis of HG models. Collectively, these findings indicate that loss of differentiation in CRC likely occurs as part of a continuum, marked by the loss of colonic epithelial transcription factors and differentiation markers including markers of LGR5+ colonic stem cells, and simultaneous acquisition of a proliferative phenotype along with markers of fetal-like intestinal stem cells and non-canonical markers including those typically expressed by mesenchymal cells.

Notably, investigation of the mechanism underlying the differential gene expression between LG and HG CRC cell lines revealed increased promoter methylation of genes suppressed in HG cell lines, including markers and drivers of colonic differentiation. This indicates stable epigenetic suppression of these genes in HG tumours, which is consistent with our finding that these markers are also suppressed in PDTOs and primary tumours derived from HG cases. Comparatively, genes downregulated in LG cell lines were not as strongly associated with promoter methylation. This likely reflects the more transient or dynamic suppression of this subset of genes in LG tumours, which were notably enriched for genes involved in highly coordinated and tightly regulated processes such as cell cycle progression.

In conclusion, while we and others have previously shown that CRC cell lines accurately reflect the major genomic alterations observed in primary CRCs^19,20,22^, we now demonstrate that CRC cells also accurately model differentiation grade. We show that differentiation loss in CRC is closely linked to loss of transcriptional programs associated with the normal colonic epithelium, such as lipid metabolism and xenobiotic detoxification, with a number of these genes displaying increased promoter methylation. We further reveal that HG tumours gain expression of cell proliferation markers as well as genes typically expressed in the fetal intestine and mesenchymal cells. Collectively, these findings may provide opportunities for specific therapeutic targeting of the poorly differentiated subset of CRC with associated poor prognosis, with CRC cell lines providing the opportune models to test such strategies.

## Methods

### Cell lines and cell culture

CRC cell lines (*n* = 84) used in this study were obtained from the ATCC or other investigators as previously described^20^ and summarised in Table S1. All cell lines were maintained at 37 °C and 5% CO_2_ in DMEM-F12 (Invitrogen), supplemented with 10% fetal calf serum (v/v), 2mM L-glutamine, 100 U/mL Penicillin and 100 µg/mL Streptomycin, all from ThermoFisher Scientific (Waltham, MA, USA). Cell line authentication was performed using the GenePrint® 10 System (Promega, Madison, WI, USA), and all cell lines for which reference short tandem repeat (STR) profiles existed produced close matches, confirming their authenticity. Cells were routinely tested for mycoplasma status using the MycoAlert Mycoplasma Detection Kit (Lonza, Basel, Switzerland). Cell line microsatellite instability status and *RAS* (*KRAS*, *NRAS*) and *BRAF* mutation status have been previously reported^19,20^.

### Generation of subcutaneous xenografts

Animal studies were performed with the approval of the Austin Health Animal Ethics Committee (AEC A2010_04036 and AEC A2015_05254). We have complied with all relevant ethical regulations for animal use. Eight-week old male Balb/*c nu/nu* mice or NOD SCID gamma (NSG) mice were obtained from the Australian Resources Centre, (ARC, Perth) and the Austin Health Bioresources Facility respectively and kept in specific pathogen free (SPF) micro-isolators. 2 × 10^6^ cells of each cell line were injected subcutaneously into the right and left flank of each animal in a 150 μL suspension consisting of a 1:1 mixture of DMEM-F12 (Invitrogen) and BD Matrigel Basement Matrix (BD Biosciences). Tumour growth was monitored every 2–3 days by caliper measurement until tumours reached a maximal size of 1 cm^3^ or the end of the experimental period (3 months), whichever occurred sooner. Xenografts were harvested at the end of the experimental period and fixed in 10% neutral buffered formalin for 24 h, then transferred to 80% ethanol. Tissues were processed and paraffin-embedded in the Department of Anatomical Pathology at Austin Health.

### Histopathological assessment

Sections from formalin-fixed and paraffin-embedded (FFPE) xenografts were stained with haematoxylin and eosin (H&E) for histopathological assessment. Two independent pathologists assessed tumour differentiation grade and mucinous features of each cell line using World Health Organization (WHO) criteria for human primary CRC^8^, which specify four grades of CRC differentiation based on the percentage of tumour exhibiting glandular structures by morphological assessment. For subsequent analyses, well (grade 1) and moderately (grade 2) differentiated tumours were analysed collectively as low-grade (LG) tumours, and poor (grade 3) and undifferentiated (grade 4) tumours were analysed collectively as high-grade (HG) tumours.

### Computation of tumour doubling time in vivo

Doubling time of the cell line xenografts was computed by growth curve modelling during the exponential phase of tumour growth using the formula (t_e_ = t_1_ + (t_2_ − t_1_) log(V_e_/V_t1_) / log(V_t2_/V_t1_)) previously described by Wu et al.^52^.

### Generation of cell blocks

For the generation of cell blocks, 1 × 10^7^ CRC cells were washed in PBS, resuspended in 150 µL of human serum, and gently mixed using a wooden stick. 150 µL of thrombin was then added to the cell suspension and mixed gently for 5 min until a clot formed. The clot was fixed in 10% formalin and processed as above.

### Immunohistochemistry (IHC)

FFPE sections (4 μm) were deparaffinised in xylene, rehydrated in graded alcohol and quenched of endogenous peroxidase activity in 3% H_2_O_2_ solution. Antigen retrieval was performed by boiling slides in 50 mM Tris-HCl (pH 8.5) (Dako) for 45 min at 100 °C. Sections were probed with the following primary antibodies: KRT20 (CK20) D9Z1Z (CST, 13063, 1:200), MUC2 996/1 (ThermoFisher, MA5-12345, 1:150), VIL1 R814 (CST, 2369S, 1:200), CDX2 D11D10 (CST, 12306S, 1:200), E-Cadherin 24E10 (CST, 3195S) by overnight incubation at 4 °C in a humidified chamber. Slides were then washed in TBS-T for 3 × 5 min and incubated with either Dako Envision anti-rabbit or anti-mouse labelled polymer-HRP (Dako) secondary antibody for 30 min. Slides were washed again in TBS-T for 3 × 5 min and then visualised by incubation with 3,3’ diaminobenzidine (DAB) chromogen for 30–60 s with colour development carefully monitored by eye, and counterstained with Mayer’s haematoxylin (Amber Scientific, Australia). Slides were then dehydrated and sealed with glass coverslips using DPX mounting solution (Sigma-Aldrich). Vimentin staining was performed by the Department of Anatomical Pathology (Austin Health) using pre-diluted Roche-Ventana Vimentin antibody, clone V9 (790-2917) on the Ventana Benchmark Ultra.

### PAS/Alcian Blue staining

FFPE sections (4 μm) were deparaffinised in xylene, rehydrated in graded alcohol and washed in distilled water for 5 min. Slides were then stained in Alcian Blue solution (pH 2.5) for 15 min, washed in running tap water for 2 min and rinsed in distilled water. The slides were then treated with 0.5% periodic acid for 5 min, washed in distilled water and stained with Schiff’s reagent for 10 min. Slides were washed again in tap water for 5 min and the nuclei stained with Mayer’s haematoxylin (Amber Scientific, Australia). Slides were then dehydrated and sealed with glass coverslips using DPX mounting solution (Sigma-Aldrich).

### Cell migration assays

CRC cell lines were seeded into a 12-well plate and allowed to reach 100% confluence. A single scratch along the middle of the well was then drawn using a P1000 pipette tip. To block cell proliferation, cells were treated with 20 μg/mL Mitomycin C (M4287-2MG, Sigma-Aldrich). Cells were then monitored for 24 h to determine the migration rate using the CKX41 inverted microscope (Olympus). The length of the wound was measured using the ImageJ software.

### Zebrafish xenotransplantation assay

Zebrafish studies were approved by the Deakin University Animal Ethics Committee (project number G22-2019). Two HG cell lines (RKO and HCT116) and 2 LG cell lines (SW948 and HRA19) were labelled with Vybrant Dil cell labelling solution at a concentration of 3 µL/mL for 20 min at 37 °C (cat#V22885, Invitrogen, ThermoFisher Scientific, Scoresby, Australia) and resuspended in DMEM media at a final density of 3 × 10^5^ cells/µL. 0.5–1 × 10^3^ cells were then microinjected into the perivitelline space (PVS) of anaesthetised zebrafish embryos (48 h post-fertilisation) as previously described^53^. Embryos with adequate transplanted cells were allowed to recover in E3 medium (5 mM NaCl, 0.17 mM KCl, 0.33 mM CaCl_2_, 0.33 mM MgSO_4_, 0.00001% methylene blue, pH 7.2) and held at 33^0^C for the duration of the study. The engraftment and visualisation of tumour cells was performed using a fluorescence microscope (Olympus MVX10 fluorescence microscope and DP72 camera using Cellsens Dimension 1.6 software). The number of metastatic cells and area of metastatic lesions in the fin tip were quantified using ImageJ analysis software with background fluorescence corrections.

### Source of RNAseq data for CRC cell lines

RNAseq data for 30 CRC cell lines were obtained from our previous study^20^. RNAseq profiles of 13 overlapping CRC cell lines were further downloaded from the Cancer Cell Line Encyclopedia database^23^. Raw reads were re-aligned to human genome build hg38 using STAR (2.7.0a)^54^ using the following parameters: --outFilterMultimapNmax 20 --alignSJoverhangMin 8 --alignSJDBoverhangMin 1 --outFilterMismatchNmax 999 --outFilterMismatchNoverReadLmax 0.05 --alignIntronMin 20 --alignIntronMax 1000000 --alignMatesGapMax 1000000 --outSAMtype BAM SortedByCoordinate --sjdbOverhang 149 --outFilterMatchNminOverLread 0.5 --outFilterScoreMinOverLread 0.5. Gene-level expression was quantified against the ENSEMBL Homo_sapiens.GRCh38.93.gtf annotation using featureCounts with a parameter to account for stranded counting (-s 2).

### Source of proteomics data for CRC cell lines

Corresponding proteomic profiles (count data) from the 30 CRC cell lines were derived from our previous study^22^.

### Methylation profiling of CRC cell lines

Methylation status of 25 CRC cell lines was profiled using Illumina 450K methylation arrays. Raw intensity data files (IDAT) were processed using the R package DNAmArray (version 2.0.0) to obtain normalised M-values. The processing pipeline included: importing raw IDAT files using read.metharray.exp; applying functional normalisation with preprocessFunnorm.DNAmArray; filtering low-quality and problematic probes with probeFiltering, imputing missing data with the imputePCA function from the missMDA package; masking ambiguous probes using probeMasking and removal of probes on sex chromosomes. This pipeline resulted in a normalised, filtered, and imputed matrix of autosomal M-values for subsequent statistical analyses.

### RNAseq analysis of CRC cell line xenografts

RNA was isolated from 8 CRC cell lines grown as xenografts and quantified using a Qubit 4.0 Fluorometer (Invitrogen, USA). Library preparation for RNAseq and next-generation sequencing was outsourced to the Australian Genome Research Facility (AGRF). Raw reads were aligned to both human genome build hg38 and mouse build mm10 with STAR (2.7.0a)^54^ as above. XenofilteR was used for filtering host from graft reads^55^. Filtered bam file was used for Gene level expression quantification against the ENSEMBL Homo_sapiens.GRCh38.93.gtf annotation using featureCounts with a parameter to account for stranded counting (-s 2).

### Quantitative real-time PCR

Total RNA was purified employing the ReliaPrep RNA isolation kit (Promega), and reverse transcribed using the High-Capacity cDNA reverse transcription kit (Applied Biosystems). Gene expression levels were determined by quantitative real-time PCR in technical triplicates using PowerSYBR green (Applied Biosystems) on a ViiA 7 Real-Time system (Life Technologies). Primers used are listed in Table S2.

### Bioinformatics analyses

Differential gene and protein expression analyses, from count data, were performed using Limma^56^ and edgeR^57^ packages. Heatmaps were generated in R using the gplots package. Elastic net regularisation was applied to reduce dimensionality and identify key genes associated with tumour grade. Gene expression data were z-score normalised, and grades were encoded as a binary outcome. A logistic regression model with elastic net penalty (alpha = 0.5) was fitted using the glmnet package in R. The optimal penalty parameter (lambda) was selected via 10-fold cross-validation (cv.glmnet). Genes with non-zero coefficients in the final model were considered informative and retained as an optimal gene list.

### RNAseq analysis of patient-derived tumour organoids

The growth of PDTOs from CRC patients (*n* = 104) used in this study has been described previously^39^. In total, 95 PDTOs originated from LG tumours and 9 PDTOs from HG tumours. RNA was isolated from patient-derived tumour organoids using QIAGEN RNeasy Mini Kit (QIAGEN, USA) and quantified using a Qubit 2.0 Fluorometer (Invitrogen, USA). Library preparation for RNAseq and next-generation sequencing was outsourced to the Ramaciotti Centre for Genomics, UNSW Sydney. Data processing and analysis were performed as for the CRC cell lines.

### TCGA dataset

The COAD and READ RNAseq count data were downloaded from the TCGA portal along with histopathology reports for each tumour. In total, *n* = 16, *n* = 186 and *n* = 58 tumours graded as GI, G2 or G3, respectively, with corresponding RNAseq data were identified.

### Gene set enrichment analysis

Gene set enrichment analysis was performed using GSEA v4.1.0 software available from the Broad Institute (https://www.gsea-msigdb.org/gsea/index.jsp)^58^. Data were analysed against the MSigDB “HALLMARKS” gene set signatures^59^, intestinal lineage signatures derived from single cell RNAseq from human intestine^24^, and “canonical”, “non-canonical” and human fetal intestinal stem cell signature described by Moorman et al.^33^.

### Statistical analyses and reproducibility

Groups were compared using Fisher’s exact test for categorical variables and unpaired *t*-tests for continuous variables unless otherwise stated, with a two-sided *P*-value < 0.05 considered statistically significant. For the xenograft study, *n* = 4 mice were used per cell line with two tumours grown per mouse (left and right flank) as per previous studies^5^. All recipient mice were age and gender matched, and no formal randomisation was performed. Analyses were performed on Prism v9.5.1 (GraphPad Software).

### Reporting summary

Further information on research design is available in the Nature Portfolio Reporting Summary linked to this article.

## Supplementary information

Supplementary Information

Supplementary Data 1

Supplementary Data 2

Description of Additional Supplementary Files

Reporting Summary

## Data Availability

RNA-Seq data from colorectal cancer cell lines utilised in Fig. 3 were accessed from the Gene Expression Omnibus (GEO) database (accession number GSE90830). https://www.ncbi.nlm.nih.gov/geo/query/acc.cgi?acc=GSE90830. RNAseq data used in Fig. 5 were downloaded from the TCGA portal (https://portal.gdc.cancer.gov/). Proteomics data used in Fig. S1 were accessed from the Supplementary data from https://pubmed.ncbi.nlm.nih.gov/28625833/. RNAseq data used in Fig. S1 were downloaded from the CCLE database (https://sites.broadinstitute.org/ccle/datasets). Cell line DNA methylation data relevant to Fig. 7 have not been publicly deposited but will be made available upon request. Total RNAseq data relevant to patient-derived tumour organoids and RNAseq data relevant to colon cancer xenografts relevant to Fig. 8 have been deposited and were given the following accession numbers, patient-derived tumour organoids: PRJNA1257965 and xenografts: GSE296173.
