# Supplementary material for: Model systems and unique biological features of high and low-grade colorectal cancer (CRC) revealed by xenografting 84 human CRC cell lines
Source: Commun Biol. 2025 Jun 5;8:875. doi: 10.1038/s42003-025-08251-0 (PMC12141688; doi:10.1038/s42003-025-08251-0)
Supplement: Supplementary file 1 — Supplementary Information [file 42003_2025_8251_MOESM1_ESM.pdf]

Figure S1. (**A**) mRNA expression of differentially expressed genes (DEGs) identified in the current study in corresponding HG (n=9) and LG (n=4) CRC cell lines profiled by the Cancer Cell Line Encyclopedia (CCLE) [25]. Plotted are expression levels of 1346 of the 1763 genes which overlapped between the 2 datasets. (**B**) Corresponding protein expression of 89 of the DEGs identified in the current study in the same 30 CRC cell lines. Proteomic data was obtained from Wang et al [22]. (**C, D**) Pearson correlations of the magnitude of differential mRNA expression between HG and LG cell lines with the corresponding magnitude of change of (**C**) mRNA or (**D**) protein expression between HG versus LG cell line of the data shown in the heatmaps in A and B respectively.

Figure S2. (A) Validation of transcription factors differentially expressed between HG and LG CRC cell lines by qPCR. Values shown are mean+SEM of a representative experiment performed in technical triplicate. (**B**, **C**) CRC cell line cell blocks with positive expression of neuroendocrine markers determined by staining for (**B**) Synaptoganin or (**C**) chromogranin. Lack of staining of (**D**) Synaptoganin and (**E**) chromogranin in HCT116 cells.

Figure S3. (A) Hallmark gene set enrichment analysis of genes differentially expressed between low and high-grade CRC cell lines. (**B**) Cell type signature GSEA showing significant enrichment of "Ki67 positive colonic cells" gene signature previously identified by Gao *et al* [33] in HG CRC cell lines. (**C**) Doubling time (mean±SEM) of LG (n=22) and HG (n=52) CRC cell lines grown as xenografts and compared using unpaired t-tests. *P<0.05.

Figure S4. Enrichment of canonical (top row) and non-canonical gene sets (bottom row) in LG vs HG (**A**) CRC cell lines grown *in vitro*, (**B**) CRC cell lines grown as xenografts, and (**C**) primary CRCs profiled by the TCGA.

Figure S5. Immunohistochemical staining of representative low grade (LG) and high grade (HG) CRC cell line cell blocks for (**A**) expression of the epithelial marker E-Cadherin and (**B**) the mesenchymal marker vimentin.

Table S1. Properties of CRC cell lines

Table S2. Table of qPCR primer sequences

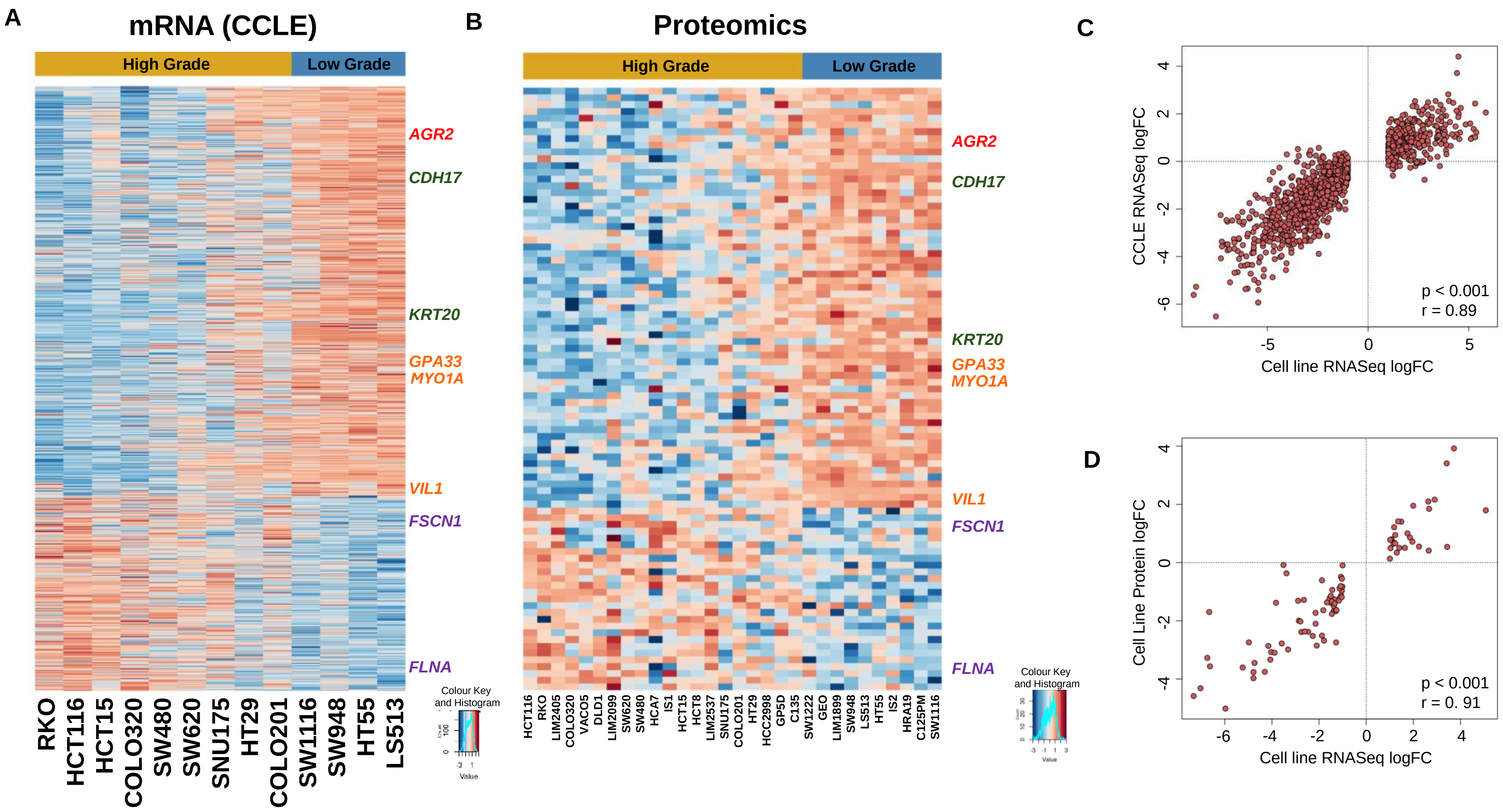

A
mRNA (CCLE)
High Grade
Low Grade
AGR2
CDH17
KRT20
GPA33
MYO1A
VIL1
FSCN1
FLNA
RKO
HCT116
HCT15
COLO320
SW480
SW620
SNU175
HT29
COLO201
SW1116
SW948
HT55
LS513
Colour Key and Histogram
Value
B
Proteomics
High Grade
Low Grade
AGR2
CDH17
KRT20
GPA33
MYO1A
VIL1
FSCN1
FLNA
HCT116
RKO
LIM2405
COLO320
VACO5
DLD1
LIM2099
SW620
SW480
HCA7
IS1
HCT15
HCT8
LIM2537
SNU175
COLO201
HT29
HCC2998
GP5D
C135
SW1222
GEO
LIM1899
SW948
LS513
HT55
IS2
HRA19
C125PM
SW1116
Colour Key and Histogram
Value
C
CCLE RNASeq logFC
Cell line RNASeq logFC
p < 0.001
r = 0.89
D
Cell Line Protein logFC
Cell line RNASeq logFC
p < 0.001
r = 0. 91

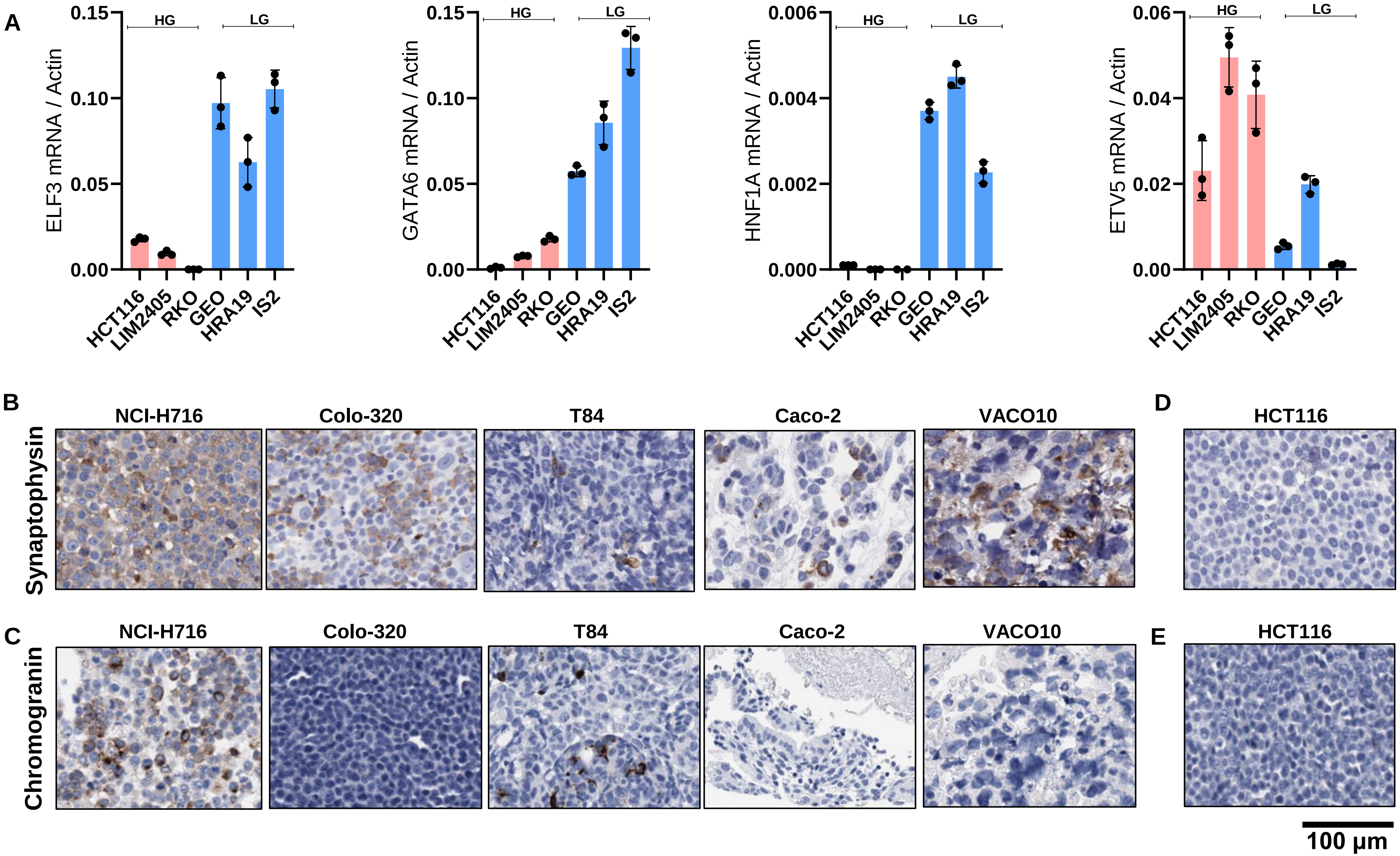
A
ELF3 mRNA / Actin
0.15
0.10
0.05
0.00
HG
LG
HCT116
LIM2405
RKO
GEO
HRA19
IS2
GATA6 mRNA / Actin
HNF1A mRNA / Actin
0.006
0.004
0.002
0.000
ETV5 mRNA / Actin
0.06
0.04
0.02
B
Synaptophysin
NCI-H716
Colo-320
T84
Caco-2
VACO10
D
HCT116
C
Chromogranin
E
100 μm

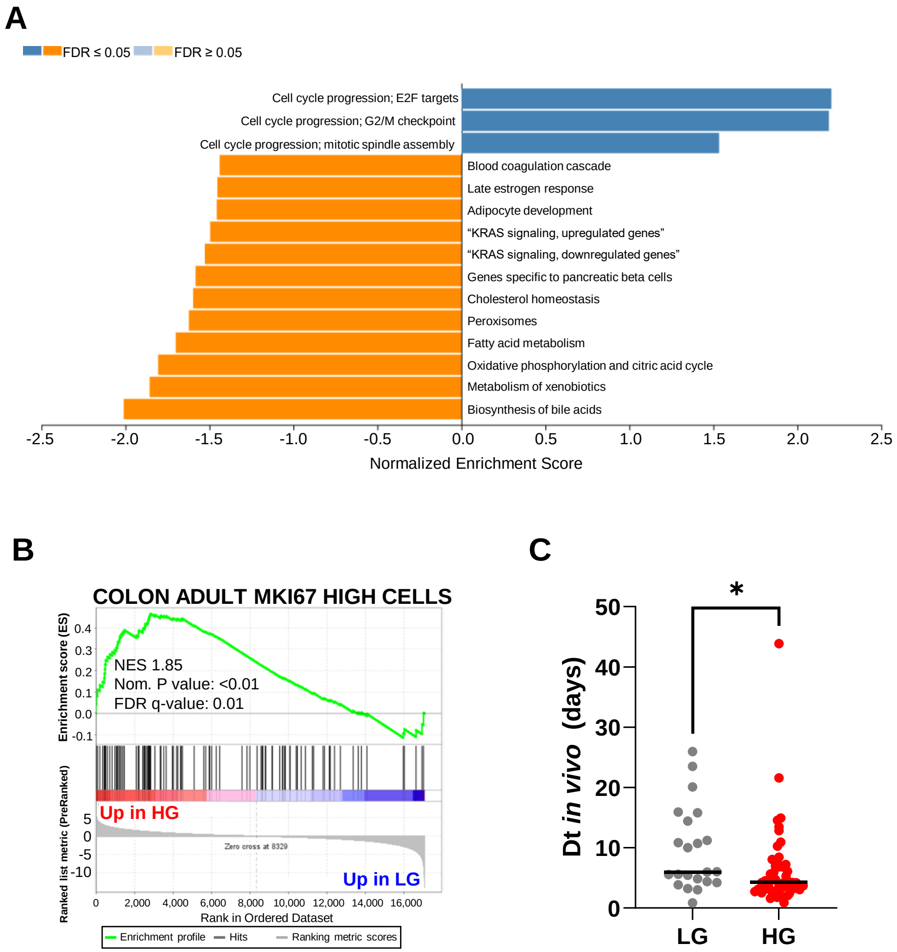
A
FDR ≤ 0.05
FDR ≥ 0.05
Cell cycle progression; E2F targets
Cell cycle progression; G2/M checkpoint
Cell cycle progression; mitotic spindle assembly
Blood coagulation cascade
Late estrogen response
Adipocyte development
"KRAS signaling, upregulated genes"
"KRAS signaling, downregulated genes"
Genes specific to pancreatic beta cells
Cholesterol homeostasis
Peroxisomes
Fatty acid metabolism
Oxidative phosphorylation and citric acid cycle
Metabolism of xenobiotics
Biosynthesis of bile acids
Normalized Enrichment Score
B
COLON ADULT MKI67 HIGH CELLS
Enrichment score (ES)
NES 1.85
Nom. P value: <0.01
FDR q-value: 0.01
Ranked list metric (PreRanked)
Up in HG
Up in LG
Zero cross at 8329
Rank in Ordered Dataset
Enrichment profile
Hits
Ranking metric scores
C
Dt *in vivo* (days)
*
LG
HG

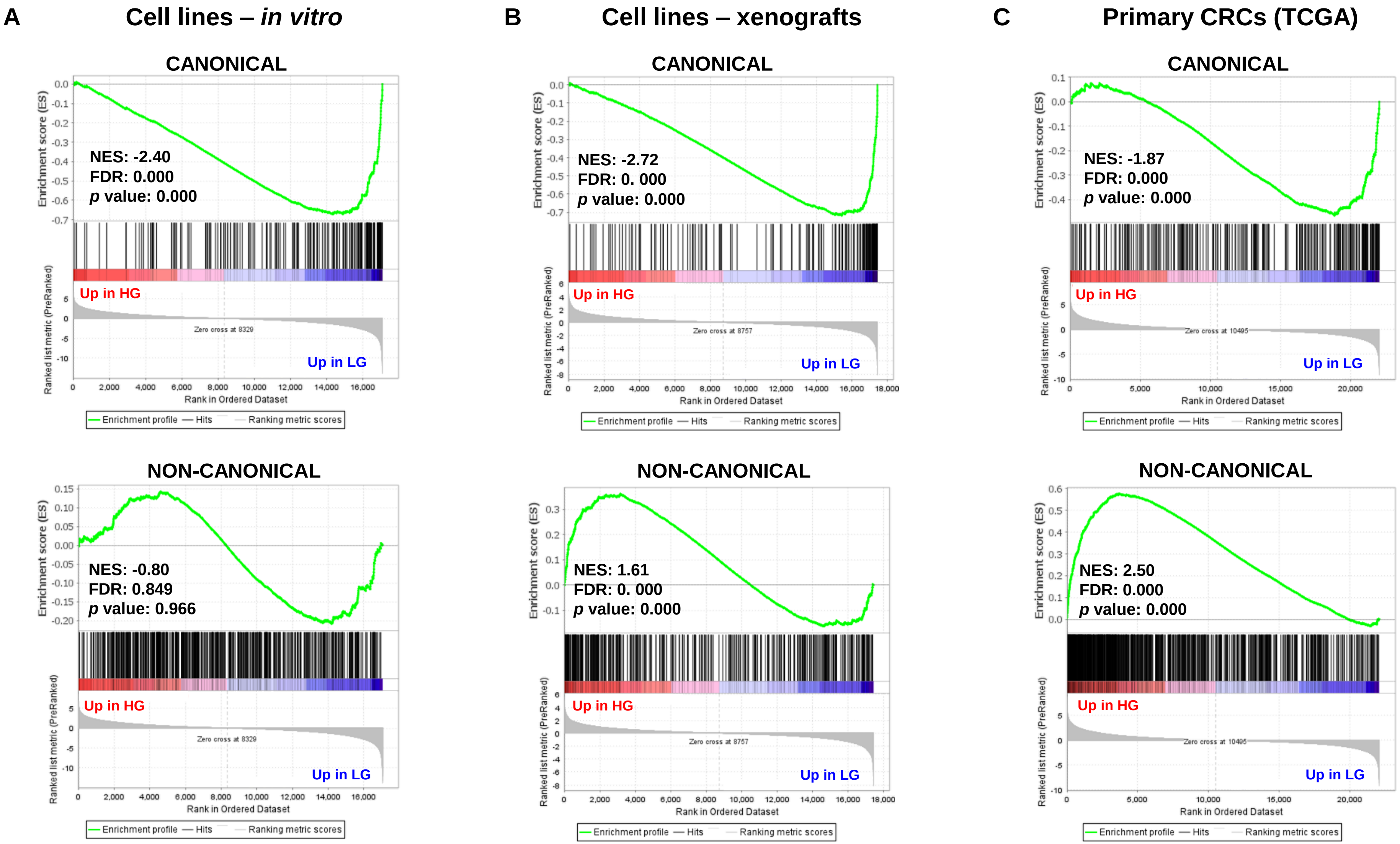
A
Cell lines – in vitro
B
Cell lines – xenografts
C
Primary CRCs (TCGA)
CANONICAL
NES: -2.40
FDR: 0.000
p value: 0.000
Up in HG
Up in LG
Zero cross at 8329
NES: -2.72
FDR: 0. 000
p value: 0.000
Zero cross at 8757
NES: -1.87
FDR: 0.000
p value: 0.000
Zero cross at 10495
NON-CANONICAL
NES: -0.80
FDR: 0.849
p value: 0.966
NES: 1.61
FDR: 0. 000
p value: 0.000
NES: 2.50
FDR: 0.000
p value: 0.000
Enrichment score (ES)
Ranked list metric (PreRanked)
Rank in Ordered Dataset
Enrichment profile
Hits
Ranking metric scores

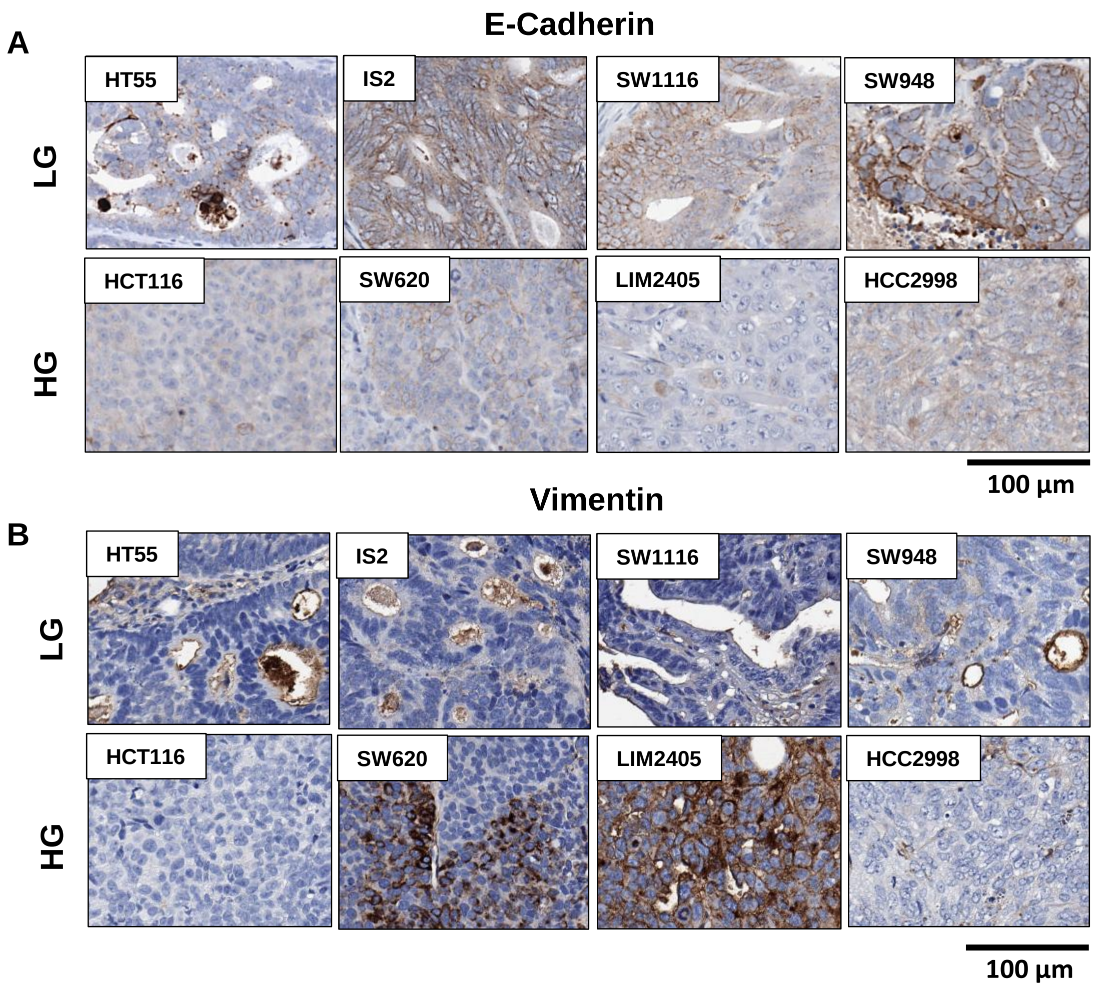

E-Cadherin
A
LG
HT55
IS2
SW1116
SW948
HG
HCT116
SW620
LIM2405
HCC2998
100 µm
Vimentin
B
LG
HT55
IS2
SW1116
SW948
HG
HCT116
SW620
LIM2405
HCC2998
100 µm

Cell line	Differentiation grade	Final Grade	MSI Status	Grade in originating tumour	CDX2	CK20	VIL1	Enterocyte markers	MUC2	PAS/AB	Goblet markers	SYN	CHR	NRAS	KRAS	BRAF	P53	Doubling time (Days)
ALA	Moderate	LG	MSS	NA	1	2	0	1	1	2	2	0	0	WT	G12D	WT	Mut	20.09
C10	Moderate	LG	MSI	MD	1	0	0	0	2	2	2	0	0	WT	WT	WT	WT	15.94
C106	Moderate	LG	MSS	MD	NA	NA	NA	NA	NA	NA	NA	NA	NA	WT	G12C	WT	Mut	15.82
C125	Moderate	LG	MSS	NA	1	1	2	2	1	0	1	0	0	WT	K117N	WT	Mut	23.50
C135	Poor	HG	MSI	NA	0	0	0	0	1	0	1	0	0	WT	WT	V600E	WT	43.86
C32	Poor	HG	MSS	WD	1	0	0	0	2	0	1	0	0	G12D	WT	WT	WT	13.48
C80	Well	LG	MSS	NA	2	1	2	2	2	2	2	0	0	WT	A146V	WT	Mut	NA
C84	Moderate	LG	MSS	PD	2	2	1	2	2	2	2	NA	NA	WT	G12A	WT	Mut	10.02
C99	Poor	HG	MSS	MD	2	1	0	1	2	0	1	0	0	WT	WT	WT	WT	21.60
CaCo2	Moderate	LG	MSS	WD	2	1	2	2	2	0	1	focal +ve	0	WT	WT	WT	Mut	10.86
CCK81	Undiff	HG	MSI	NA	2	1	0	1	2	0	1	0	0	WT	WT	WT	Mut	6.97
Co115	Undiff	HG	MSI	PD	0	0	0	0	0	0	0	0	0	WT	WT	V600E	ND	4.21
Colo201	Poor	HG	MSS	PD	0	1	1	2	1	0	1	0	0	WT	WT	V600E	Mut	5.88
Colo205	Undiff	HG	MSS	PD	0	0	0	0	1	0	1	0	0	WT	WT	V600E	Mut	5.63
Colo320	Undiff	HG	MSS	PD	2	0	0	0	0	0	0	focal +ve	0	WT	WT	WT	Mut	3.45
Colo678	Undiff	HG	MSS	NA	NA	NA	NA	NA	NA	NA	NA	0	0	WT	G12D	WT	WT	NA
CX1	Undiff	HG	MSS	NA	2	2	1	2	2	0	1	NA	0	WT	WT	V600E	Mut	7.10
DiFi	Undiff	HG	MSS	NA	1	1	0	1	0	0	0	0	0	WT	WT	WT	ND	3.83
DLD1	Undiff	HG	MSI	NA	2	1	0	1	2	0	1	0	0	WT	G13D	WT	WT	2.86
GEO	Moderate	LG	MSS	NA	1	0	1	1	1	1	2	0	0	WT	G12A	WT	ND	3.01
GP2D	Undiff	HG	MSI	PD	1	2	0	1	2	0	1	0	0	WT	G12D	WT	WT	12.84
GP5D	Poor	HG	MSI	PD	2	0	0	0	2	0	1	0	0	WT	G12D	WT	WT	14.57
HCA46	Moderate	LG	MSS	PD	1	1	2	2	1	0	1	0	0	WT	WT	WT	Mut	5.54
HCA7	Undiff	HG	MSI	MD	0	0	0	0	1	0	1	0	0	WT	WT	WT	Mut	8.43
HCC2998	Undiff	HG	MSS	NA	0	0	0	0	0	0	0	0	0	WT	A146T	WT	Mut	6.78
HCT116	Undiff	HG	MSI	NA	0	0	0	0	0	0	0	0	0	WT	G13D	WT	WT	0.91
HCT15	Undiff	HG	MSI	NA	1	0	0	0	0	0	0	0	0	WT	G13D	WT	WT	4.71
HCT8	Undiff	HG	MSI	MD	1	0	0	0	0	0	0	0	0	WT	G13D	WT	WT	6.08
HDC54	Poor	HG	MSS	MD	0	1	0	1	0	1	1	0	0	WT	WT	WT	Mut	4.22
HDC82	Undiff	HG	MSS	MD	0	2	0	1	0	0	0	0	0	WT	WT	WT	Mut	3.96
HDC87	Poor	HG	MSS	MD	1	0	0	0	2	0	1	0	0	WT	G12V	WT	WT	10.28
HDC90	Poor	HG	MSS	MD	NA	NA	NA	NA	NA	NA	NA	0	0	WT	G12R	WT	Mut	4.17
HRA19	Moderate	LG	MSS	WD	1	1	1	2	0	1	1	0	NA	WT	WT	WT	Mut	25.95
HT115	Poor	HG	MSS	NA	1	2	1	2	2	0	1	0	0	WT	WT	WT	Mut	14.91
HT29	Poor	HG	MSS	W-MD	0	2	0	1	2	2	2	0	0	WT	WT	V600E	Mut	6.98
HT55	Moderate	LG	MSS	NA	2	1	1	2	1	1	2	NA	0	WT	WT	WT	Mut	4.39
HuTu80	Undiff	HG	MSS	NA	0	0	0	0	1	0	1	NA	0	WT	WT	WT	ND	2.07
IS1	Undiff	HG	MSS	NA	1	1	0	1	1	0	1	0	0	ND	G12D	WT	Mut	10.91
IS2	Moderate	LG	MSS	NA	2	2	2	2	2	2	2	0	0	ND	G12D	WT	Mut	10.72
IS3	Moderate	LG	MSS	NA	1	2	1	2	2	2	2	0	0	ND	G12D	WT	Mut	4.84
KM12	Undiff	HG	MSI	NA	0	0	0	0	2	0	1	0	0	WT	WT	WT	Mut	4.11
LIM1215	Undiff	HG	MSI	PD	0	0	0	0	0	0	0	0	0	WT	WT	WT	WT	1.76
LIM1863	Poor	HG	MSS	PD	0	1	1	2	0	0	0	0	0	WT	WT	WT	Mut	5.32

Cell line	Differentiation grade	Final Grade	MSI Status	Grade in originating tumour	CDX2	CK20	VIL1	Enterocyte markers	MUC2	PAS/AB	Goblet markers	SYN	CHR	NRAS	KRAS	BRAF	P53	Doubling time (Days)
LIM1899	Moderate	LG	MSI	MD	2	0	2	1	2	2	2	0	0	WT	G12A (het)	WT	WT	3.86
LIM2099	Poor	HG	MSS	NA	1	2	2	2	1	1	2	0	0	WT	G12C	WT	WT	8.06
LIM2405	Undiff	HG	MSI	PD	0	0	0	0	0	0	0	0	0	WT	WT	V600E	WT	2.07
LIM2537	Undiff	HG	MSI	NA	0	0	0	2	0	0	0	0		WT	WT	V600E	Mut	2.19
LIM2550	Undiff	HG	MSI	NA	0	0	0	2	2	0	1	0	0	WT	Q61K (het)	WT	Mut	6.83
LIM2551	Undiff	HG	MSI	NA	0	0	0	2	0	0	0	0	0	WT	WT	V600E	Mut	4.70
LOVO	Poor	HG	MSI	NA	0	0	0	2	2	0	1	0	0	WT	G13D	WT	WT	3.13
LS1034	Moderate	LG	MSS	M-PD	2	2	0	1	2	0	1	NA	NA	WT	A146T	WT	ND	6.01
LS174T	Moderate	LG	MSI	W-MD	0	0	1	1	0	1	1	0	0	WT	G12D	WT	WT	0.85
LS180	Poor	HG	MSI	WD	1	0	1	1	1	2	2	NA	NA	WT	G12D	WT	WT	2.51
LS411	Poor	HG	MSI	PD	0	0	0	0	1	0	1	0	0	WT	WT	V600E	Mut	3.66
LS513	Moderate	LG	MSS	NA	1	1	0	1	0	1	1	0	0	WT	G12D	WT	WT	3.21
NCIH -716	Undiff	HG	MSS	PD	0	0	0	0	2	0	1	Positive	Positive	WT	WT	WT	Mut	4.51
NCIH-747	Moderate	LG	MSS	W-MD	NA	NA	NA	NA	NA	NA	NA	0	0	WT	G13D	WT	Mut	NA
RKO	Undiff	HG	MSI	NA	0	0	0	0	0	0	0	0	0	WT	WT	V600E	WT	3.04
RW2982	Undiff	HG	MSS	W-MD	0	0	0	0	0	0	0	0	0	WT	Q61R	WT	Mut	3.14
RW7213	Moderate	LG	MSS	W-MD	2	2	0	1	2	2	2	0	0	WT	G12C	WT	Mut	14.43
SKCO1	Undiff	HG	MSS	NA	1	2	1	2	1	0	1	0	0	WT	G12V	WT	WT	8.84 (NSG)
SNU175	Poor	HG	MSI	NA	0	0	0	0	2	2	2	0	0	WT	WT	WT	WT	5.75 (NSG)
SNU283	Well	LG	MSS	MD	2	1	2	2	2	2	2	NA	NA	WT	WT	WT	WT	6.04 (NSG)
SNUC1	Undiff	HG	MSS	MD	2	2	0	1	2	0	1	0	0	WT	WT	WT	Mut	8.97 (NSG)
SNUC2B	Poor	HG	MSI	MD	0	0	0	0	1	1	2	0	0	WT	G12D	WT	Mut	3.86
SNUC4	Undiff	HG	MSI	MD	2	0	0	0	2	0	1	0	0	WT	WT	WT	WT	3.12
SW1116	Well	LG	MSS	MD	2	2	2	2	2	1	2	0	0	WT	G12A	WT	Mut	11.21
SW1222	Moderate	LG	MSS	MD	2	0	1	1	2	1	2	0	0	WT	A146V	WT	WT	6.14 (NSG)
SW1417	Undiff	HG	MSS	NA	0	0	0	0	1	0	1	0	0	WT	WT	V600E	Mut	NA (NSG)
SW1463	Moderate	LG	MSS	NA	1	1	2	2	1	1	2	NA	NA	WT	G12C	WT	Mut	5.45
SW403	Moderate	LG	MSS	PD	2	2	2	2	1	1	2	0	0	WT	G12V	WT	Mut	4.21
SW48	Undiff	HG	MSI	PD	0	0	0	0	0	0	0	0	0	WT	WT	WT	WT	1.59
SW480	Undiff	HG	MSS	PD	0	0	0	0	1	0	1	0	0	WT	G12V	WT	Mut	6.76
SW620	Undiff	HG	MSS	PD	0	0	0	0	0	0	0	0	0	WT	G12V	WT	Mut	4.34
SW837	Poor	HG	MSS	PD	0	0	0	0	0	0	0	0	0	WT	G12C	WT	Mut	3.25
SW948	Moderate	LG	MSS	PD	2	1	2	2	0	2	1	0	0	WT	Q61L	WT	Mut	5.96
T84	Undiff	HG	MSS	NA	0	0	0	0	1	0	1	rare cells +ve	rare cells +ve	WT	G13D	WT	WT	2.39
TC71	Undiff	HG	MSI	NA	0	0	0	0	1	2	2	0	NA	ND	G12D	WT	Mut	3.69
V9P	Undiff	HG	MSS	NA	0	0	0	0	0	0	0	0	0	WT	WT	WT	Mut	4.29
VACO10	Moderate	LG	MSS	MD	2	2	1	2	1	1	2	Positive	0	WT	Q61R	WT	Mut	5.59
VACO432	Undiff	HG	MSI	NA	0	1	0	1	1	0	1	NA	NA	WT	WT	V600E	ND	2.71
VACO4S	Undiff	HG	MSS	NA	NA	NA	NA	NA	NA	NA	NA	0	0	WT	G12V	WT	Mut	NA (NSG)
VACO5	Undiff	HG	MSI	PD	1	0	0	0	2	0	1	0	0	WT	WT	V600E	ND	2.74
WiDr	Poor	HG	MSS	NA	0	2	0	1	2	2	2	0	0	WT	WT	V600E	Mut	7.21

Gene	Forward	Reverse
ANXA1	5'-CTAAGCGAAACAATGCACAGC-3'	5'-CCTCCTCAAGGTGACCTGTAA-3'
ASCL2	5'-CTGGTGAACTTGGGCTTCC-3'	5'-CAGCGTCTCCACCTTGCT-3'
ELF3	5'-GGGGCCAAAAGAAAAAGAAC-3'	5'-AACTTGTAGACGAGTCGCCG-3'
EPHB2	5'-AGAAACGCTAATGGACTCCACT-3'	5'-GTGCGGATCGTGTTCATGTT-3'
ETV5	5'-CAGTCAACTTCAAGAGGCTTGG-3'	5'-TGCTCATGGCTACAAGACGAC-3'
GATA6	5'-CAAACCAGGAAACGAAAACC-3'	5'-AAGAGGTGGAAGTTGGAGTCA-3'
HNF1A	5'-GTGTGGCGAAGATGGTCAAGT-3'	5'-CTGTGGGATGTTGTGCTGCT-3'
ICAM1	5'-GTATGAACTGAGCAATGTGCAAG-3'	5'-GTTCCACCCGTTCTGGAGTC-3'
LGR5	5'-CTGCTCCGACCTGGGGCTCT-3'	5'-GCGGAGACTGGGCAGGGGAT-3'
MRAS	5'-TTCCTCATCGTCTACTCCGTC-3'	5'-AGGATCATCGGGAATGACTCC-3'
OLFM4	5'-ACCTTTCCCGTGGACAGAGT-3'	5'-TGGACATATTCCCTCACTTTGGA-3'
TSPAN4	5'-CTGTCCTCTTCCTTCCCGTC-3'	5'-CGTGTAGGCGAAGAAGAGGA-3'
