# Supplementary material for: Model systems and unique biological features of high and low-grade colorectal cancer (CRC) revealed by xenografting 84 human CRC cell lines
Source: Commun Biol. 2025 Jun 5;8:875. doi: 10.1038/s42003-025-08251-0 (PMC12141688; doi:10.1038/s42003-025-08251-0)
Supplement: Supplementary file 4 — Description of Additional Supplementary Files [file 42003_2025_8251_MOESM4_ESM.docx]

**Description of Additional Supplementary Files**

File name: Supplementary Data 1

Description: Reduced gene list for grade-associated transcriptomic profiling

File name: Supplementary Data 2

Description: Source data file
