# Supplementary material for: Model systems and unique biological features of high and low-grade colorectal cancer (CRC) revealed by xenografting 84 human CRC cell lines
Source: Commun Biol. 2025 Jun 5;8:875. doi: 10.1038/s42003-025-08251-0 (PMC12141688; doi:10.1038/s42003-025-08251-0)
Supplement: Supplementary file 5 — Reporting Summary [file 42003_2025_8251_MOESM5_ESM.pdf]

nature portfolio

Corresponding author(s): Mariadon, John
Sieber, Oliver

Last updated by author(s): 16/05/2025

Reporting Summary

Nature Portfolio wishes to improve the reproducibility of the work that we publish. This form provides structure for consistency and transparency in reporting. For further information on Nature Portfolio policies, see our Editorial Policies and the Editorial Policy Checklist.

Statistics

For all statistical analyses, confirm that the following items are present in the figure legend, table legend, main text, or Methods section.

n/a	Confirmed	
☐	☒	The exact sample size (*n*) for each experimental group/condition, given as a discrete number and unit of measurement
☐	☒	A statement on whether measurements were taken from distinct samples or whether the same sample was measured repeatedly
☐	☒	The statistical test(s) used AND whether they are one- or two-sided<br>*Only common tests should be described solely by name; describe more complex techniques in the Methods section.*
☒	☐	A description of all covariates tested
☒	☐	A description of any assumptions or corrections, such as tests of normality and adjustment for multiple comparisons
☐	☒	A full description of the statistical parameters including central tendency (e.g. means) or other basic estimates (e.g. regression coefficient) AND variation (e.g. standard deviation) or associated estimates of uncertainty (e.g. confidence intervals)
☒	☐	For null hypothesis testing, the test statistic (e.g. *F*, *t*, *r*) with confidence intervals, effect sizes, degrees of freedom and *P* value noted<br>*Give P values as exact values whenever suitable.*
☒	☐	For Bayesian analysis, information on the choice of priors and Markov chain Monte Carlo settings
☒	☐	For hierarchical and complex designs, identification of the appropriate level for tests and full reporting of outcomes
☒	☐	Estimates of effect sizes (e.g. Cohen's *d*, Pearson's *r*), indicating how they were calculated

Our web collection on statistics for biologists contains articles on many of the points above.

Software and code

Policy information about availability of computer code

Data collection

RNAseq profiles of 12 CRC cell lines were downloaded from the Cancer Cell line encyclopedia database. The COAD and READ RNAseq count data were downloaded from the TCGA portal along with histopathology reports for each tumour.

Data analysis

Analysis of RNAseq data from cell lines: Raw reads were re-aligned to human genome build hg38 using STAR (2.7.0a) 26 using the following parameters: --outFilterMultimapNmax 20 --alignSJoverhangMin 8 --alignSJDBoverhangMin 1 --outFilterMismatchNmax 999 --outFilterMismatchNoverReadLmax 0.05 --alignIntronMin 20 --alignIntronMax 1000000 --alignMatesGapMax 1000000 --outSAMtype BAM SortedByCoordinate --sjdbOverhang 149 --outFilterMatchNminOverLread 0.5 --outFilterScoreMinOverLread 0.5. Gene level expression was quantified against the ENSEMBL Homo_sapiens.GRCh38.93.gtf annotation using featureCounts with a parameter to account for stranded counting (-s 2).

RNAseq analysis of xenografts: Raw reads were aligned to both human genome build hg38 and mouse build mm10 with STAR (2.7.0a) 26 as above. XenofilteR was used for filtering host from graft reads. Filtered bam file was used for Gene level expression quantification against the ENSEMBL Homo_sapiens.GRCh38.93.gtf annotation using featureCounts with a parameter to account for stranded counting (-s 2).

Differential gene and protein expression analyses, from count data were performed using Limma and edgeR packages. Heatmaps were generated in R using the gplots package. Elastic net regularization was applied to reduce dimensionality and identify key genes associated with tumor grade.

Methylation status of 25 CRC cell lines were profiled using Illumina 450K methylation arrays. Raw intensity data files (IDAT) were processed using the R package DNAmArray (version 2.0.0) to obtain normalized M-values. The processing pipeline included: importing raw IDAT files using read.metharray.exp; applying functional normalization with preprocessFunnorm.DNAmArray; filtering low-quality and problematic

probes with probeFiltering, imputing missing data with the imputePCA function from the missMDA package; masking ambiguous probes using probeMasking and removal of probes on sex chromosomes. This pipeline resulted in a normalized, filtered, and imputed matrix of autosomal M-values for subsequent statistical analyses.

For manuscripts utilizing custom algorithms or software that are central to the research but not yet described in published literature, software must be made available to editors and reviewers. We strongly encourage code deposition in a community repository (e.g. GitHub). See the Nature Portfolio guidelines for submitting code & software for further information.

Data

Policy information about availability of data

All manuscripts must include a data availability statement. This statement should provide the following information, where applicable:

- Accession codes, unique identifiers, or web links for publicly available datasets
- A description of any restrictions on data availability
- For clinical datasets or third party data, please ensure that the statement adheres to our policy

Data availability statement provided in manuscript.

Research involving human participants, their data, or biological material

Policy information about studies with human participants or human data. See also policy information about sex, gender (identity/presentation), and sexual orientation and race, ethnicity and racism.

Reporting on sex and gender	N/A
Reporting on race, ethnicity, or other socially relevant groupings	N/A
Population characteristics	N/A
Recruitment	N/A
Ethics oversight	N/A

Note that full information on the approval of the study protocol must also be provided in the manuscript.

Field-specific reporting

Please select the one below that is the best fit for your research. If you are not sure, read the appropriate sections before making your selection.

☒ Life sciences ☐ Behavioural & social sciences ☐ Ecological, evolutionary & environmental sciences

For a reference copy of the document with all sections, see nature.com/documents/nr-reporting-summary-flat.pdf

Life sciences study design

All studies must disclose on these points even when the disclosure is negative.

Sample size	Sample size calculations were not performed.
Data exclusions	No data have been excluded.
Replication	All attempts to replicate data have been successful.
Randomization	N/A
Blinding	N/A

Reporting for specific materials, systems and methods

We require information from authors about some types of materials, experimental systems and methods used in many studies. Here, indicate whether each material, system or method listed is relevant to your study. If you are not sure if a list item applies to your research, read the appropriate section before selecting a response.

Materials & experimental systems

n/a	Involved in the study	
☐	☒	Antibodies
☐	☒	Eukaryotic cell lines
☒	☐	Palaeontology and archaeology
☐	☒	Animals and other organisms
☒	☐	Clinical data
☒	☐	Dual use research of concern
☒	☐	Plants

Methods

n/a	Involved in the study	
☒	☐	ChIP-seq
☒	☐	Flow cytometry
☒	☐	MRI-based neuroimaging

Antibodies

Antibodies used	KRT20 (CK20) D9Z1Z (CST, 13063, 1:200), MUC2 996/1 (ThermoFisher, MA5-12345, 1:150), VIL1 R814 (CST, 2369S, 1:200), CDX2 D11D10 (CST, 12306S, 1:200), E-Cadherin 24E10 (CST, 3195S)
Validation	Expression of all proteins detected using these antibodies in cell lines correlated strongly with corresponding mRNA expression.

Eukaryotic cell lines

Policy information about cell lines and Sex and Gender in Research

Cell line source(s)	CRC cell lines (n=84) used in this study were obtained from the ATCC or other investigators as previously described (Reference 20 in manuscript).
Authentication	Cell line authentication was performed using the GenePrint® 10 System (Promega, Madison, WI, USA), and all cell lines for which reference short tandem repeat (STR) profiles existed produced close matches confirming their authenticity.
Mycoplasma contamination	Cells were routinely tested for mycoplasma status using the MycoAlert Mycoplasma Detection Kit (Lonza, Basel, Switzerland).
Commonly misidentified lines (See ICLAC register)	N/A

Animals and other research organisms

Policy information about studies involving animals; ARRIVE guidelines recommended for reporting animal research, and Sex and Gender in Research

Laboratory animals	Eight-week old male Balb/c nu/nu mice or NOD SCID gamma (NSG) mice
Wild animals	N/A
Reporting on sex	Only male mice were used.
Field-collected samples	N/A
Ethics oversight	Animal studies were performed with the approval of the Austin Health Animal Ethics Committee (AEC A2010_04036 and AEC A2015_05254).

Note that full information on the approval of the study protocol must also be provided in the manuscript.

Plants

Seed stocks	N/A
Novel plant genotypes	N/A
Authentication	N/A
